# Fabrication of Sub-10 μm Microvias Using Nanosecond UV Laser Drilling and a Sacrificial Metal Barrier Layer for Advanced Fine-Pitch Packaging

**DOI:** 10.3390/mi17060709

**Published:** 2026-06-10

**Authors:** Nam-Son Park, Tae-Young Lee, Kyoung-Min Kim, Hyun-Jin Nam, Hee-Chul Lee

**Affiliations:** 1 Department of Advanced Materials Engineering, Tech University of Korea, Siheung 15073, Republic of Korea; parkns@tukorea.ac.kr; 2 Cooperative Research Facilities, Tech University of Korea, Siheung 15073, Republic of Korea; lty1226@tukorea.ac.kr (T.-Y.L.); kkm386@tukorea.ac.kr (K.-M.K.); 3 ICT Device Packaging Research Center, Korea Electronics Technology Institute, Siheung 15073, Republic of Korea; hjnam1203@keti.re.kr

**Keywords:** microvia, UV laser drilling, SMBL, ABF, surface roughness, signal integrity, plasma desmear, interconnect reliability

## Abstract

Advanced packaging requires high-density interconnects with sub-10 μm design rules; however, conventional processes involving laser drilling and plasma desmearing increase dielectric surface roughness and degrade signal performance. A nanosecond ultraviolet (ns-UV) laser microvia process using a sacrificial metal barrier layer (SMBL) was developed to enable sub-10 μm via formation while preserving dielectric surface integrity. A Cu SMBL was introduced to block debris redeposition during laser irradiation and shield the dielectric from ion bombardment during plasma processing. By optimizing laser power, shot count, and SMBL thickness, approximately 8 μm microvias were formed in 10 μm thick Ajinomoto Build-up Film (ABF). The Cu SMBL facilitated heat dissipation, reducing the heat-affected zone and limiting lateral widening at the via entrance, resulting in improved via geometry and higher taper. The dielectric surface roughness increased significantly (>80 nm) when no SMBL was used during processing, whereas it remained nearly constant with the SMBL (Ra: 6.36 → 6.43 nm), thereby reducing current scattering at high frequencies. Adhesion of 0.46 kgf/cm was maintained after quick-via-pull testing without mechanical interlocking, with no interfacial separation observed, confirming reliable interconnect formation. Therefore, the SMBL process enables precise microvia fabrication and low-loss interconnects for high-frequency packaging.

## 1. Introduction

Advanced packaging improves chip performance by enabling circuit miniaturization in line with Moore’s law [1,2]. However, further scaling of single chips is limited by physical and economic constraints. Therefore, heterogeneous integration using chiplets and 2.1D/2.5D/3D packaging has been widely adopted [3]. To achieve high input/output (I/O) density in organic interposers and build-up substrates for high-performance computing, aggressive scaling to 2/2 μm line/space (L/S) and sub-10 μm interconnect design rules is required [4,5,6,7].

As the operating frequency of next-generation substrates reaches tens of GHz, signal integrity (SI) becomes a major design challenge [8,9,10,11]. At high frequencies, the skin effect confines current flow to a thin region near the conductor surface, making electrical performance highly sensitive to the morphology of the conductor–dielectric interface. When the skin depth decreases to the nanometer scale, surface asperities disrupt current distribution, inducing electromagnetic scattering and increasing the effective current path length, thereby increasing insertion loss. Consequently, substrates with lower surface roughness exhibit reduced signal attenuation, confirming that surface morphology governs conductor loss in high-frequency interconnects. Therefore, maintaining a nanometer-scale smooth dielectric surface during microvia fabrication is essential for achieving high signal integrity in advanced packaging substrates [12,13,14,15,16,17].

However, conventional CO_2_ laser drilling generates excessive heat due to its long wavelength (9.3–10.6 μm), which limits fine via formation and damages the dielectric surface [18]. In addition, wet desmearing removes polymer faster than fillers, causing polymer recession and filler protrusion. This condition produces a rough surface that complicates lithography, increases seed layer overetching, and degrades interfacial quality, making it difficult to scale microvias and maintain high-frequency performance [19].

The key challenge in microvia fabrication is achieving sub-10 μm vias while maintaining a smooth dielectric surface. However, ultraviolet (UV) laser drilling alone cannot prevent surface damage during desmearing. Therefore, a process that can physically protect the dielectric surface is required.

Picosecond UV laser processing has been widely investigated for ultra-fine microvia fabrication because its shorter pulse duration reduces thermal diffusion and heat-affected-zone formation compared with nanosecond laser processing. Previous studies have demonstrated that picosecond UV lasers can fabricate microvias with diameters of several micrometers in ABF and other organic dielectric materials. Therefore, picosecond UV laser technology is a promising route for next-generation fine-pitch packaging [20].

However, the practical choice between picosecond UV and nanosecond UV laser systems depends not only on via quality but also on manufacturing throughput, equipment cost, process stability, maintenance cost, and compatibility with existing package-substrate production lines. Recent developments in high-power and high-repetition-rate picosecond UV lasers have improved their throughput and cost-performance ratio. Nevertheless, nanosecond UV laser systems remain attractive for industrial substrate manufacturing because they are already widely adopted, robust, relatively cost-effective, and compatible with high-speed large-area processing. The purpose of this study is therefore not to replace picosecond UV laser processing, but to extend the capability of industrially mature nanosecond UV laser drilling by introducing a sacrificial metal barrier layer (SMBL). The SMBL suppresses debris redeposition, reduces laser-induced surface damage, and protects the ABF surface during plasma desmearing, thereby enabling sub-10 μm microvia fabrication using a nanosecond UV laser process [21,22,23].

## 2. Materials and Methods

### 2.1. Substrate Preparation

**Figure 1:**
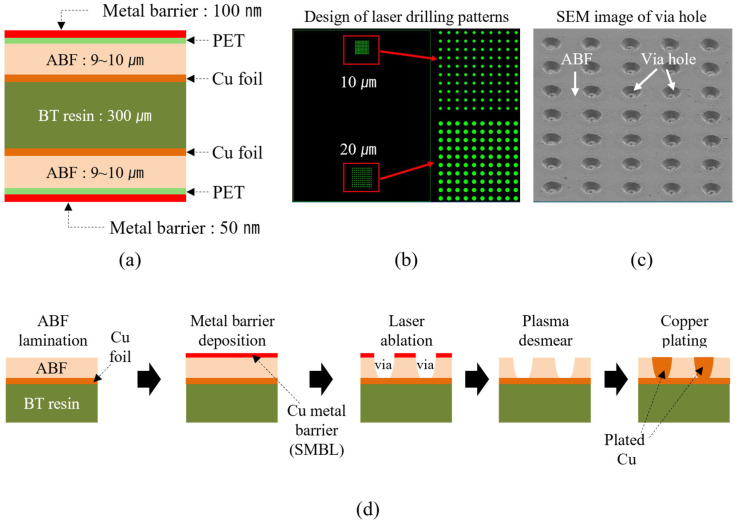
Test substrate structure with (**a**) Cu sacrificial metal barrier layer (SMBL), (**b**) laser drilling array design, (**c**) scanning electron microscopy (SEM) micrograph of fabricated microvias, and (**d**) schematic of the SMBL-assisted process flow [24].

Figure 1a shows the schematic of the test substrate structure. Ajinomoto Build-up Film (ABF-GX-T62, Ajinomoto Fine-Techno Co., Inc., Kawasaki, Japan), a 10 μm thick nanofiller-reinforced epoxy dielectric, was used as the dielectric layer. The test substrate consisted of a 0.3 mm thick Bismaleimide Triazine (BT) core with 7 μm thick Cu foils laminated on both sides. The ABF was first vacuum-laminated onto the substrate and then fully cured in a convection oven. Subsequently, an SMBL was deposited on the ABF surface by a direct current (DC) magnetron sputtering system (JSVISS-6C, Jesagi Hankook Ltd., Siheung, Republic of Korea). Figure 1b shows the laser drilling array design, and Figure 1c shows a scanning electron microscopy (SEM, Apreo 2 S, Thermo Fisher Scientific, Waltham, MA, USA) image of the fabricated microvias. Figure 1d presents a schematic of the integrated process sequence for microvia formation on an ABF/BT substrate employing the SMBL.

Two different SMBL materials, Cu and Ni–Cr, were compared to evaluate the influence of material properties on microvia formation and surface integrity. Cu was selected as a reference material due to its high thermal conductivity and compatibility with Cu-based interconnect layers, whereas Ni–Cr was chosen for its excellent chemical resistance to wet etching during SMBL removal. This comparison enables systematic assessment of the roles of thermal properties and chemical stability in SMBL-assisted processing. The SMBL thickness was varied from 50 to 200 nm. As shown in Figure 1d, microvias were formed by ns-UV laser drilling through the SMBL, followed by plasma desmearing to remove residues at the via bottom. The SMBL was subsequently removed by wet chemical etching (Cu: H_2_SO_4_–H_2_O_2_; Ni–Cr: CAN/HNO_3_). After SMBL removal, Ti/Cu seed layers (~50 nm Ti/~300 nm Cu) were deposited by sputtering, followed by Cu electroplating to complete microvia metallization. To ensure sufficient interfacial adhesion between the ABF and deposited SMBL during subsequent UV laser drilling and desmearing, vacuum degassing and Ar ion-beam pretreatment were applied prior to SMBL deposition.

### 2.2. Nanosecond UV Laser Drilling and Desmearing Process

A 355 nm ns-UV laser (4500U Special, EO Technics Co., Ltd., Anyang, Republic of Korea) was employed for microvia drilling. In preliminary tests, the laser was operated at a repetition rate of 50 kHz with a pulse width of 25 ns, and the focal position was set at a Z-offset of +0.3 mm. For the experimental proper, the laser was operated at a pulse width of 25 ns and a repetition rate of 50 to 300 kHz in punch mode. The laser power ranged from 0.10 to 0.45 W, corresponding to a fluence of 0.66 to 17.9 J/cm^2^. The focal position was controlled by a Z-offset, and an offset of +0.05 μm yielded optimal via taper and roundness. Plasma desmearing was conducted using reactive ion etching (RIE) for 420 s at 5000 W with an Ar/O_2_/CF_4_ gas mixture, effectively removing polymer residues and debris. The Cu SMBL was subsequently chemically etched, followed by ultrasonic cleaning in deionized (DI) water to remove residual particles.

### 2.3. Characterization and Reliability Evaluation

Microvia geometry was characterized using optical microscopy (OM) and focused ion beam SEM (FIB-SEM; Quanta 3D FEG, Thermo Fisher Scientific, MA, USA). Surface roughness was quantified by atomic force microscopy (AFM) over a scan area of 5 × 5 μm using the standardized roughness parameters *R*_a_, *R*_z_, and *S*_a_ to monitor surface changes after each process step. In this study, *R*_a_ (ISO 4287) represents the arithmetic average of the absolute profile height deviations from the mean line, whereas *R*_z_ (ISO 4287) corresponds to the total profile height defined as the sum of the maximum peak height (*R*_p_) and the maximum valley depth (*R*_v_). In addition, *S*_a_ (ISO 25178) represents the three-dimensional extension of *R*_a_ and corresponds to the areal average of the absolute height deviations from the mean plane [25,26]. These parameters provide complementary information on both line-based and area-based surface characteristics, enabling a comprehensive evaluation of surface morphology evolution during processing.

**Figure 2:**
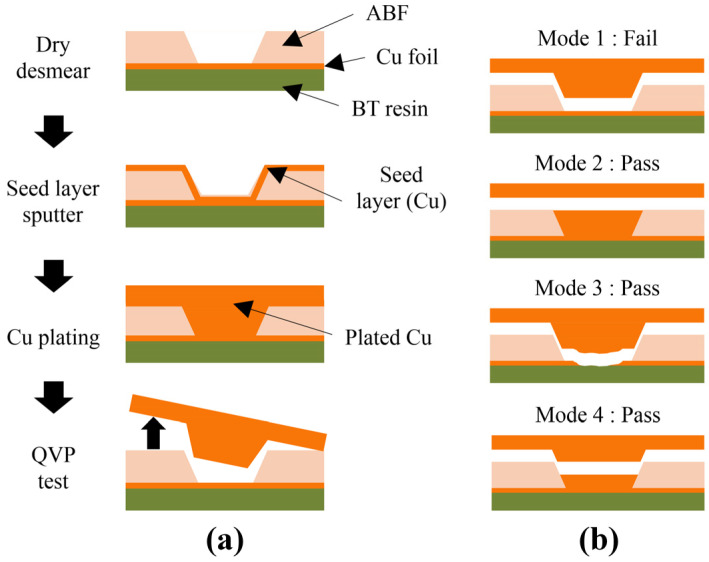
(**a**) Specimen preparation for quick-via-pull (QVP) testing and (**b**) anticipated failure modes [28].

The via-bottom interfacial reliability was evaluated using the QVP test, as illustrated in Figure 2. Test specimens were prepared by sequential plasma desmear, seed-layer sputtering, Cu electroplating, and a 30 min heat treatment at 180 °C, followed by mechanical loading to induce interfacial failure. As shown in Figure 2b, four distinct failure modes are anticipated, where Mode 1 corresponds to interfacial delamination and is classified as a failure, whereas Modes 2–4 indicate successful interfacial bonding with different deformation characteristics [27].

## 3. Results and Discussion

### 3.1. Effects of Laser Parameters and SMBL Thickness on Microvia Geometry and Stability

A preliminary evaluation was conducted to define laser process conditions for sub-10 μm microvia formation using an ns-UV laser.

**Figure 3:**
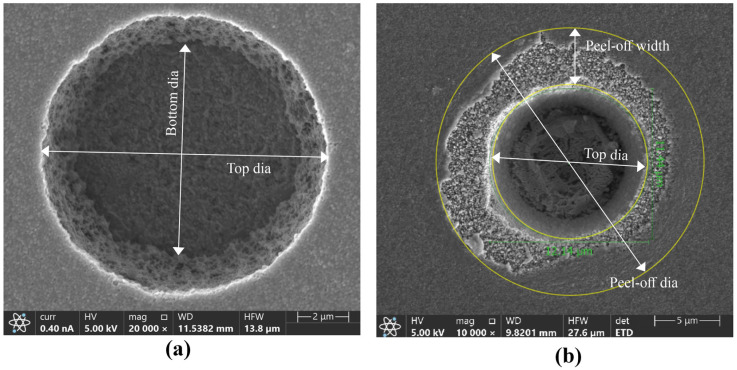
Definition of (**a**) taper and (**b**) SMBL peel-off diameter and width.

As seen in Figure 3a, the via profile was characterized by measuring the top diameter (Top dia) and bottom diameter (Bottom dia) from the SEM micrographs. These parameters were used to evaluate via geometry and taper, which are critical for the reliability of fine-pitch interconnections. In Figure 3b, the SMBL peel-off region around the via was analyzed. The peel-off diameter (Peel-off dia) was defined as the outer boundary of the affected region, whereas the peel-off width (Peel-off width) was determined as the radial distance between the via edge (Top dia) and the outer peel-off boundary. This metric quantified the extent of SMBL surface damage or delamination induced during laser drilling and subsequent desmear. These geometrical definitions enabled quantitative comparison of via quality and surface integrity under different process conditions.

**Figure 4:**
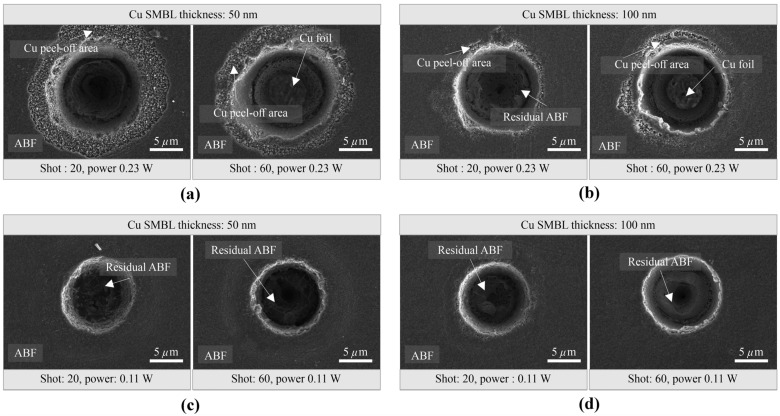
SEM micrographs of laser-drilled microvias on ABF with different Cu SMBL thicknesses and laser conditions: (**a**) 50 nm, 0.23 W; (**b**) 100 nm, 0.23 W; (**c**) 50 nm, 0.11 W; (**d**) 100 nm, 0.11 W (shots: 20 and 60; repetition rate: 50 kHz; Z-offset: +0.3 mm).

As shown in Figure 4, at a laser power of 0.11 W and 20–60 shots, via diameters of approximately 9.5–10.5 μm were obtained with minimal SMBL peel-off. Increasing the shot count to 60–80 shots enlarged the via diameter to 11–12.5 μm and initiated SMBL peel-off at the via edge. At higher power (0.20–0.23 W) and ≥80 shots, the via diameter further increased to 13–13.5 μm, accompanied by severe delamination due to excessive thermal loading. SMBL thickness strongly influenced this behavior. With a 50 nm SMBL, sub-10 μm vias were achieved only within a narrow range (≤0.13 W and ≤40 shots), and SMBL peel-off rapidly increased outside this process window. By contrast, a 100 nm SMBL maintained via diameters below 10 μm up to approximately 0.15 W and 60 shots while suppressing delamination. This improvement resulted from enhanced heat spreading and mechanical stability, which reduced laser-induced delamination. Accordingly, the feasible process window for sub-10 μm microvia formation was defined as 0.11–0.15 W laser power and 20–60 shots with a 100 nm SMBL.

The observed dependence of via diameter and delamination behavior on SMBL thickness can be attributed to differences in thermal transport during ns-UV laser irradiation. In thin metallic films, heat conduction plays a critical role in dissipating localized thermal energy induced by laser pulses.

According to Fourier’s law of heat conduction,

(1) q=−k∇T,

where q is the heat flux, k is the thermal conductivity, and ∇T is the temperature gradient. A thicker SMBL provides a larger effective heat-conduction pathway, enabling more efficient lateral heat spreading and reducing localized temperature rise.

For thin films, the in-plane heat spreading capability can be approximated using the sheet thermal conductance:

(2) $$G_{s}=k\cdot t$$

where $G_{s}$ is the sheet conductance and t is the film thickness. Increasing the SMBL thickness from 50 nm to 100 nm effectively doubles heat conduction, improving thermal dissipation.

The characteristic thermal diffusion length during a laser pulse can be estimated as

(3) $$L_{d}=\sqrt{\alpha \tau }$$

where α is the thermal diffusivity and τ is the pulse duration. For nanosecond laser pulses, the diffusion length is limited, resulting in strong localized heating. Therefore, insufficient heat spreading in thinner SMBL (50 nm) leads to higher peak temperatures, promoting interfacial stress and delamination.

By contrast, thicker SMBL (100 nm) enhance lateral heat spreading, reducing thermal gradients and suppressing laser-induced delamination. This explains the broader process window observed for sub-10 μm via formation with increased SMBL thickness.

And thermal effusivity (*e*) can be defined as

(4) $$e=\sqrt{k\rho c_{p}}$$

where k is the thermal conductivity, ρ is the density, and $c_{p}$ is the specific heat capacity. The estimated thermal effusivity of the Cu SMBL is $e_{Cu}\approx 36,900\;Wm^{-2}K^{-1}s^{1/2}$, whereas that of the ABF dielectric is $e_{ABF}\approx 600\;Wm^{-2}K^{-1}s^{1/2}$. Thus, the thermal effusivity of Cu is approximately 62 times higher than that of ABF. This substantial difference indicates that under transient pulsed heating, the Cu SMBL can absorb and redistribute heat significantly more effectively than the underlying ABF dielectric. Assuming a first-order approximation for a bi-material interface subjected to a pulsed heat input, the fraction of the interfacial heat flux absorbed by each material scales with its thermal effusivity. Therefore, the Cu SMBL accommodates approximately 98% of the transient interfacial heat flux during each laser pulse, leaving the ABF substantially less exposed to abrupt temperature excursions.

Furthermore, we evaluated the areal heat capacity (*C_A_*) of the 100 nm Cu SMBL, which is given by

(5) $$C_{A}=\rho c_{p}d$$

where d is the SMBL thickness. For a 100 nm Cu SMBL, the areal heat capacity is approximately $0.34\;Jm^{-2}K^{-1}.$ In comparison, the areal heat capacity of the thermally affected zone within the ABF is estimated at approximately $0.15\;Jm^{-2}K^{-1}.$ Consequently, during a single laser pulse, the areal heat capacity of the Cu SMBL is approximately 2.3 times greater than that of the corresponding thermally affected ABF region.

These quantitative estimates demonstrate that the Cu SMBL functions as a transient thermal capacitor during ns-UV laser irradiation. It effectively absorbs and dissipates the laser-induced thermal load before any significant temperature rise propagates into the ABF dielectric. As a result, the SMBL mitigates transient thermal excursions within the ABF, suppresses lateral thermal decomposition, minimizes heat-affected-zone (HAZ) formation, and reduces thermomechanical stress at the SMBL/ABF interface. This transient thermal-shielding mechanism strongly corroborates the experimentally observed reductions in via-opening enlargement, SMBL peel-off, and dielectric surface degradation.

**Figure 5:**
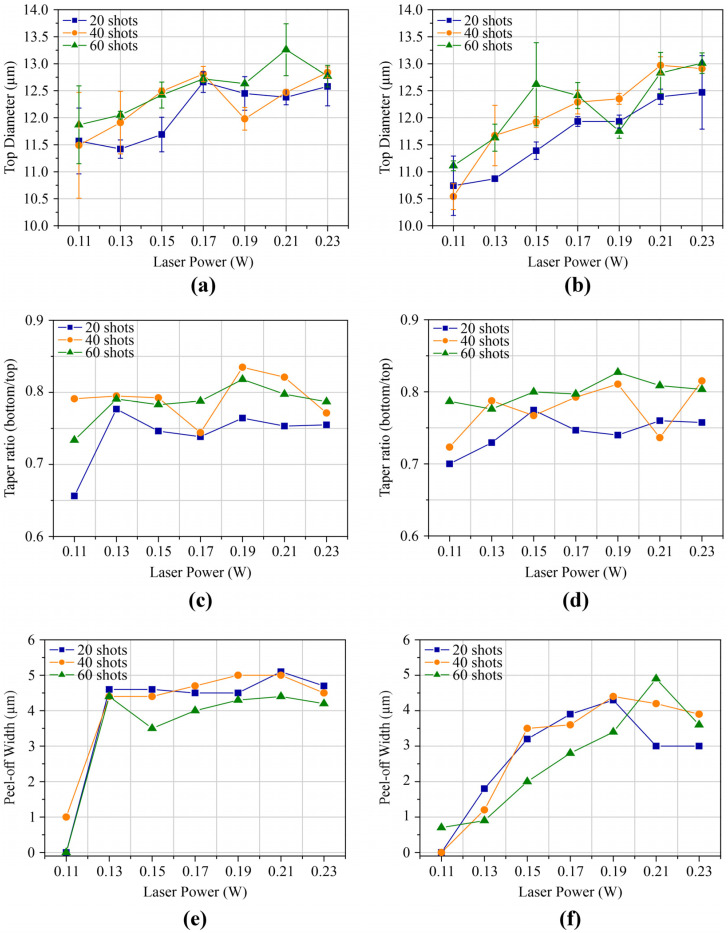
Top diameter (**a**,**b**), taper (**c**,**d**), and SMBL peel-off width (**e**,**f**) versus laser power for different shot counts (20, 40, and 60) with 50 nm (**a**,**c**,**e**) and 100 nm (**b**,**d**,**f**) Cu SMBL (50 kHz, Z-offset: +0.3 mm).

Figure 5 shows how the top diameter (a, b), taper (c, d), and SMBL peel-off width (e, f) varied with laser power and shot count for 50 and 100 nm SMBLs. Under the same y-axis scale, the 100 nm SMBL produced smaller and more uniform top diameters than the 50 nm SMBL. For both thicknesses, increasing the shot count from 20 to 60 increased the top diameter due to cumulative energy input. However, this increase was smaller for the 100 nm SMBL, indicating stronger resistance to laser-induced expansion. Increasing the SMBL thickness from 50 to 100 nm suppressed excessive top-opening enlargement by spreading laser-induced heat and blocking debris redeposition. As a result, a combination of low laser power and a thicker SMBL enabled tighter control of via size and stable formation of sub-10 μm microvias.

Taper was evaluated from top-view SEM images (Figure 5c). The SMBL peel-off diameter was defined as the outer diameter of the region where the SMBL was removed around the via by laser energy, and the peel-off width was calculated as (peel-off diameter − top diameter)/2, as shown in Figure 5e. Variations in top diameter were directly reflected in taper (Figure 5c,d). The taper increased with shot count, consistent with increased sidewall material removal under higher cumulative energy. The 100 nm SMBL exhibited a narrower taper distribution across laser power, whereas the 50 nm SMBL exhibited larger variation, especially at higher shot counts. The peel-off width (Figure 5e,f) increased with both laser power and shot count. For the 50 nm SMBL, it increased from approximately 0–1 μm at 0.11 W to 4.5–5 μm at ≥0.21 W. By contrast, the 100 nm SMBL limited the peel-off width to about 1–2 μm at low power and 3–4 μm at high power. Increasing laser power from 0.11 to 0.23 W enlarged the top diameter from 10–11 μm to 13–13.5 μm, and the taper followed this trend. Overall, the 100 nm SMBL reduced peel-off width by approximately 30–50% and stabilized via geometry.

**Figure 6:**
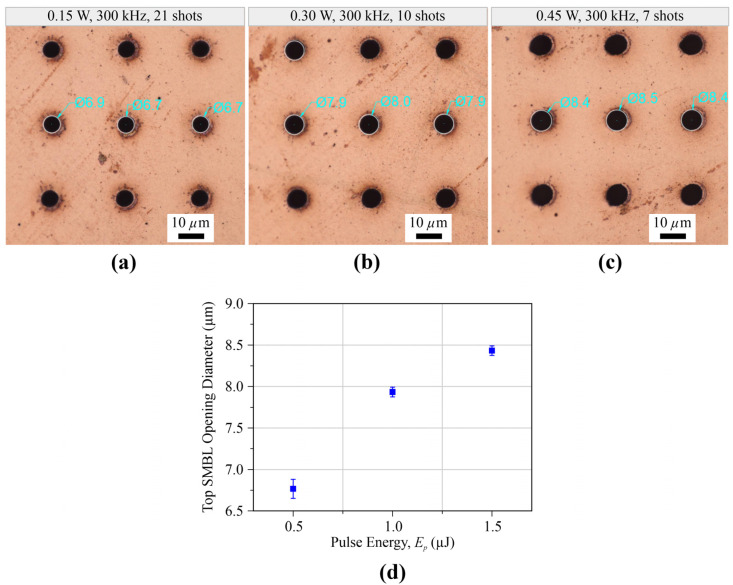
(**a**–**c**) Top-view images of SMBL openings and (**d**) opening diameter as a function of pulse energy for a 100 nm Cu SMBL (300 kHz, Z-offset = 0 mm).

To isolate the effect of energy delivery per pulse, the total laser fluence was maintained at a similar level by adjusting the number of shots, while the pulse energy was systematically varied at a fixed repetition rate of 300 kHz. As shown in Figure 6a–c, increasing the laser power from 0.15 to 0.45 W increased the opening diameter from approximately 6.8 to ~8.5 μm, despite reducing the number of shots. This behavior indicates that higher pulse energy removed a larger volume of material per pulse and generated stronger local heating, which drove lateral material removal and widened the via entrance. This trend is clearly reflected in Figure 6d: as the pulse energy decreased from 1.5 to 0.5 μJ, the opening diameter correspondingly decreased from approximately 8.5 to 6.8 μm. At lower pulse energy, each pulse deposited less energy, leading to reduced material removal per pulse and weaker lateral heat diffusion. As a result, lateral expansion at the SMBL surface was suppressed. Therefore, even at similar total fluence, distributing the energy over multiple low-energy pulses limited lateral ablation and yielded smaller, more precisely defined via openings.

**Figure 7:**
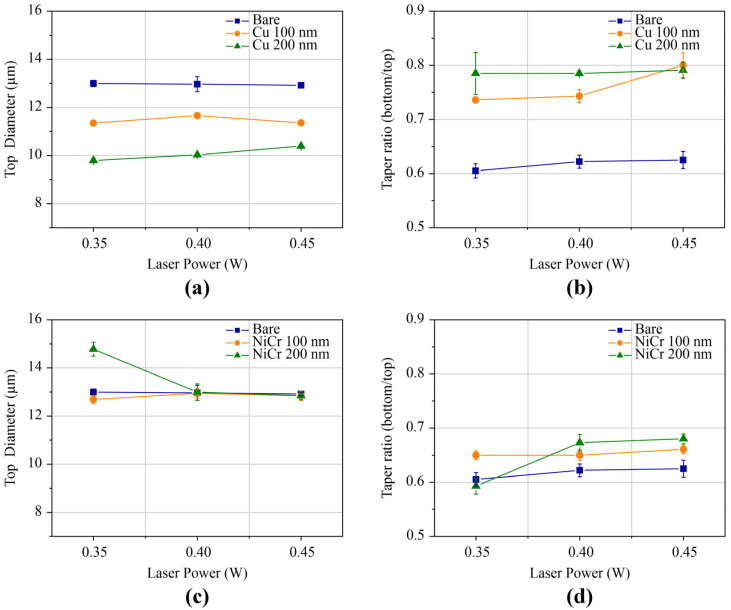
Top diameter, bottom diameter, and taper as a function of laser power for the (**a**,**b**) Cu-series and (**c**,**d**) NiCr-series samples (BARE, 100 nm, 200 nm; 300 kHz, Z-offset: +0.05 mm).

Figure 7 shows how SMBL material and thickness redistribute laser energy and determine the resulting via geometry. For the Cu series (Figure 7a), the top diameter remained nearly constant in the BARE sample but decreased when a Cu SMBL was applied. This reduction became more pronounced with increasing Cu thickness, with the 200 nm layer producing the smallest openings. The Cu SMBL spread heat laterally, reducing surface energy input and suppressing lateral ablation at the via entrance.

As shown in Figure 7b, the taper ratio (bottom/top) was significantly higher in the Cu SMBL samples than in the BARE sample and increased with laser power. This increase in taper resulted from the asymmetric change in top and bottom dimensions induced by the SMBL. The Cu SMBL suppressed lateral expansion at the via entrance, thereby minimizing the increase in top diameter during both laser drilling and subsequent plasma desmear. At the same time, under identical plasma desmearing conditions, the via bottom was more effectively opened, leading to an increase in bottom diameter. The increased taper with the Cu SMBL stemmed from suppressed top expansion and enhanced bottom opening under an identical desmearing recipe.

For the NiCr series (Figure 7c), the top diameter did not decrease with increasing thickness. In particular, the 200 nm NiCr layer produced a larger opening (~14.8 μm at ~0.35 W) than the Cu SMBL even at low power. This observation indicates that NiCr did not effectively suppress lateral material removal at low pulse energy, as the laser energy remained localized near the surface, causing strong surface heating and lateral ablation at the via entrance. As a result, all the NiCr samples exhibited lower taper ratios (~59–67%) than the Cu SMBL (Figure 7b,d), indicating that lateral expansion dominated over vertical material removal. By contrast, the Cu SMBL suppressed top expansion and directed more energy toward the via bottom, resulting in smaller top openings, higher taper, and more stable via profiles. This difference resulted from the thermal properties of the SMBL materials: Cu spread heat and reduced surface energy concentration, whereas NiCr retained heat at the surface, enhancing lateral ablation and limiting bottom etching.

As shown in Figure 7a, the Cu SMBL (~400 W/m·K) rapidly conducted heat away from the laser irradiation zone, reducing the local temperature at the via entrance. This limited the thermal decomposition of the dielectric and suppressed lateral material removal, resulting in well-defined openings in the range of 8 to 11 μm.

By contrast, as shown in Figure 7c, the Ni–Cr SMBL (~15 W/m·K) did not effectively conduct heat, causing the deposited laser energy to remain localized near the surface. This led to rapid temperature rise, accelerated dielectric decomposition, and enhanced lateral ablation, resulting in excessive top expansion with diameters exceeding 15 μm.

**Figure 8:**
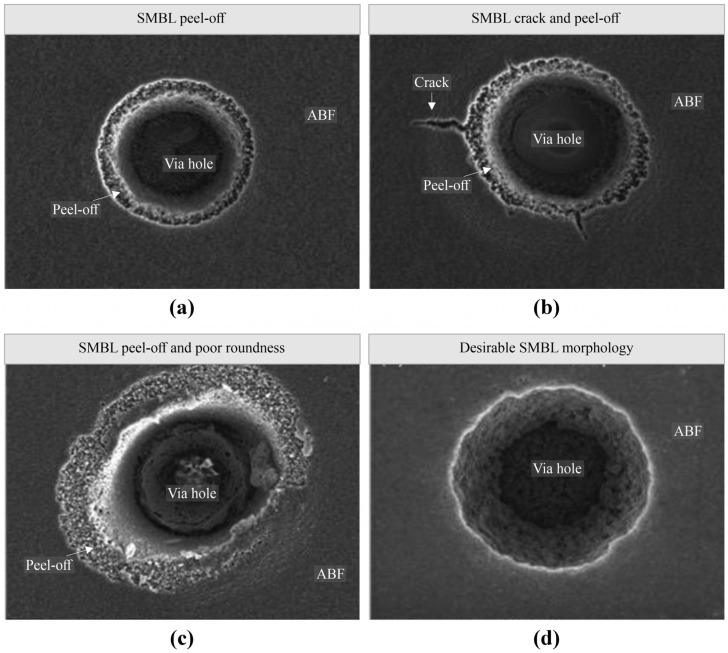
Failure modes under nonoptimized conditions: (**a**) SMBL peeling, (**b**) radial cracking with peeling, (**c**) severe peel-off with poor roundness, and (**d**) optimized via shape.

In ns-UV laser processing with an SMBL, improper optimization of the SMBL material, thickness, or laser parameters can lead to excessive heat accumulation and induce SMBL delamination and radial cracking, as summarized in Figure 8. These failures occurred when the deposited laser energy was not efficiently dissipated, leading to a rapid rise in local temperature and the build-up of thermomechanical stress at the SMBL/dielectric interface. These results demonstrated that the SMBL controlled via formation by governing heat flow during laser irradiation. A high thermal conductivity SMBL would spread heat laterally, reduce the peak temperature at the irradiation site, and stabilize material removal by preventing localized overheating.

### 3.2. Surface Integrity Analysis

**Figure 9:**
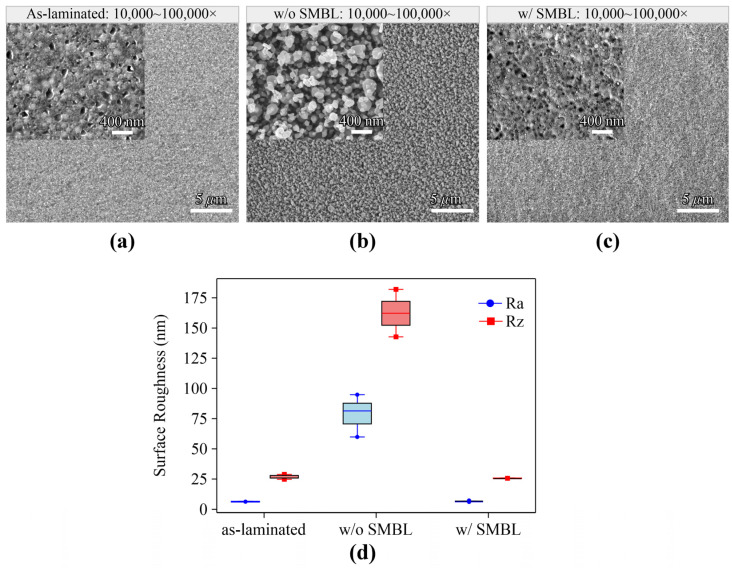
SEM images (**a**–**c**) and surface roughness (**d**) of ABF surfaces: (**a**) as-laminated, (**b**) without (w/o) SMBL, and (**c**) with (w/) SMBL.

**Figure 10:**
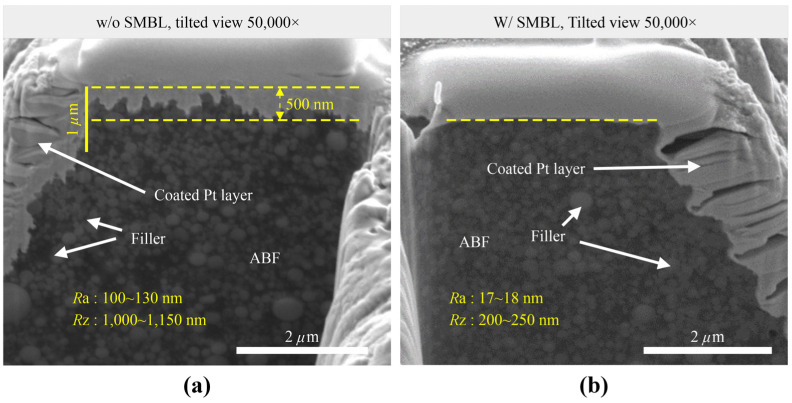
FIB and SEM of specimen (**a**) w/o and (**b**) w/ Cu SMBL.

Figure 9 and Figure 10 present the surface morphology and subsurface microstructure of ABF dielectric layers characterized using SEM, AFM, and FIB cross-sectional analysis. The as-laminated ABF exhibited a smooth, featureless surface (*R*_a_ = 6.36 nm, *R*_z_ = 28.94 nm), which served as an optimal baseline for high-frequency signal transmission (Figure 9a). Without SMBL protection, the surface roughness increased by over one order of magnitude after laser drilling and O_2_ + Ar + CF_4_ RIE plasma desmearing (*R*_a_ ≈ 78.75 nm, *R*_z_ ≈ 162.57 nm). SEM imaging revealed preferential etching of the epoxy matrix by reactive fluorine and oxygen species, leaving SiO_2_ fillers protruding from the recessed surface (Figure 9b). This differential etch selectivity created an uneven topology that is detrimental to metallization uniformity. With SMBL, the surface remained statistically equivalent to the as-laminated condition (*R*_a_ ≈ 6.43 nm, *R*_z_ ≈ 25.95 nm), confirming effective plasma shielding (Figure 9c).

FIB cross-sections (Figure 10) corroborated these findings. Without SMBL, peak-to-valley heights exceeded 500 nm with exposed fillers (*R*_a_ = 100–130 nm, *R*_z_ = 1000–1150 nm). With SMBL, the surface remained planar with embedded fillers (*R*_a_ = 17–18 nm, *R*_z_ = 200–250 nm), representing a 5–6× reduction in roughness, which is essential for uniform seed layer deposition.

**Figure 11:**
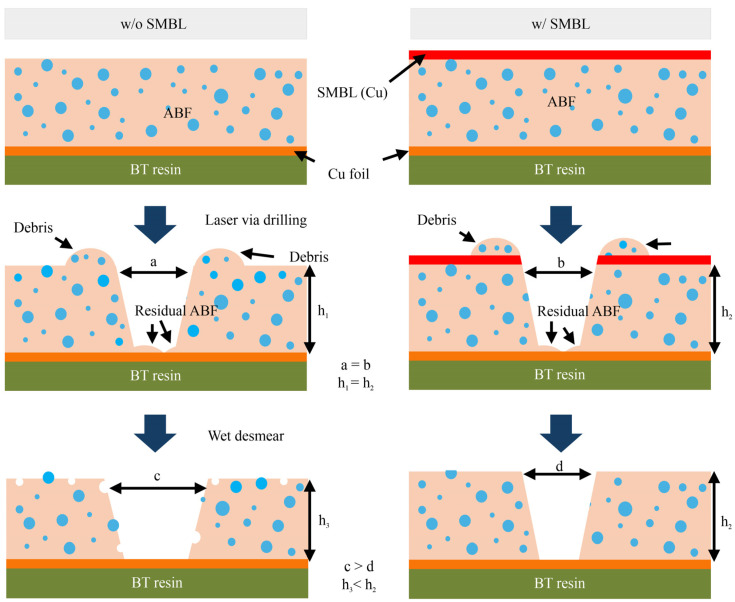
Surface integrity comparison: process schematic, (**left**) w/o and (**right**) w/ SMBL.

Figure 11 schematically illustrates the evolution of surface integrity during via fabrication with and without Cu SMBL. The differences in via morphology between the two conditions can be explained sequentially through the process steps, as follows.

• Initial structure: The left pathway (a) represents the conventional stack with ABF directly laminated on a BT resin core. The right pathway (b) incorporates a Cu SMBL deposited on the ABF surface prior to processing.
• After laser via drilling: Both configurations produced comparable via geometries with equivalent aperture dimensions (a = b) and residual ABF thicknesses (h_1_ = h_2_). Carbonaceous debris formed along the sidewalls in both cases. However, the Cu SMBL provided lateral heat dissipation, reducing thermal damage to the surrounding ABF region.
• After desmearing treatment: Without SMBL, the O_2_ + Ar + CF_4_ RIE plasma attacked the entire exposed ABF surface. Reactive species preferentially etched the epoxy matrix, while SiO_2_ fillers resisted oxidation, resulting in filler protrusion and surface roughening. The via aperture expanded (c > a), and residual thickness decreased (h_3_ < h_1_).

With SMBL, the copper layer shielded the ABF field surface from plasma exposure.

Desmearing occurred selectively within the via cavity only. Consequently, the via dimension remained stable (d ≈ b), residual thickness was preserved (h_2_), and the surface retained its as-laminated smoothness with fully embedded fillers. The dimensional relationships (c > d, h_3_ < h_2_) quantitatively demonstrated that Cu SMBL prevented both lateral via expansion and vertical material loss while maintaining nanometer-scale surface planarity, which is essential for subsequent metallization.

### 3.3. Adhesion Strength and Interconnect Reliability Evaluation

Forming reliable Cu interconnects on ultra-smooth dielectric surfaces is challenging because reduced roughness lowers mechanical interlocking at the interface. Therefore, adhesion and via reliability were evaluated using a 90° peel test and a Quick Via Pull (QVP) test. The test samples were prepared by depositing a 100 nm Cu SMBL, followed by laser drilling and plasma desmear, removing the SMBL, sputtering a Ti/Cu seed layer (100/300 nm), electroplating Cu to 20 μm, and heat-treating at 190 °C for 1 h.

The smooth dielectric surface exhibits a stable adhesion strength of 0.46 kgf/cm. The measured values were consistent across 10 measurement points (0.38–0.52 kgf/cm), indicating uniform interfacial bonding and excellent process reproducibility. Notably, these results demonstrated that stable adhesion can be achieved even on an ultra-smooth surface without relying on conventional roughness-induced mechanical interlocking. This behavior suggests that the SMBL-assisted process enabled the formation of a chemically stable and uniformly bonded interface. In particular, the Ti/Cu seed layer facilitated strong interfacial bonding, whereas the preserved smooth dielectric surface promoted intimate and continuous contact across the interface. As a result, reliable adhesion was maintained despite the absence of microscale anchoring structures typically required in conventional roughened surfaces. Consequently, the proposed process achieved robust metallization and vertical interconnect formation while preserving nanoscale surface integrity. This capability provides a practical pathway to simultaneously achieve fine-pitch microvia scaling and the low-loss interconnect performance required for next-generation high-frequency packaging through ns-UV laser processing.

**Figure 12:**
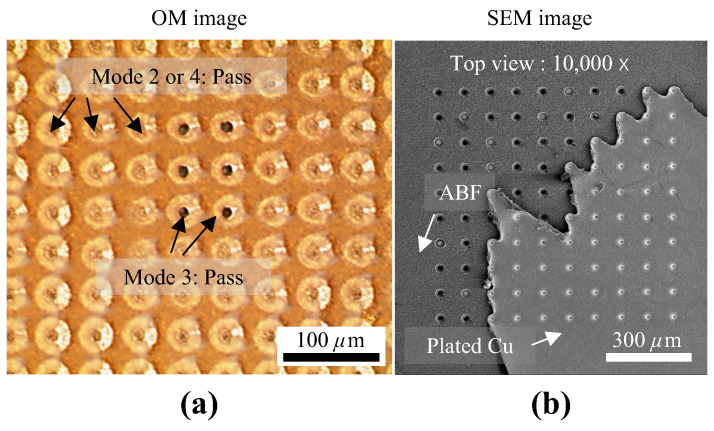
QVP test for evaluating via-bottom interfacial reliability: (**a**) optical microscopy (OM) and (**b**) SEM micrographs.

As mentioned in Section 2.3, four distinct failure modes were anticipated for the QVP test (Figure 2b). The OM micrographs in Figure 12a revealed that most vias exhibited Mode 2 and Mode 3 behavior, indicating stable interconnect formation. Furthermore, the SEM micrograph in Figure 12b presents representative via morphologies consistent with these failure modes.

**Figure 13:**
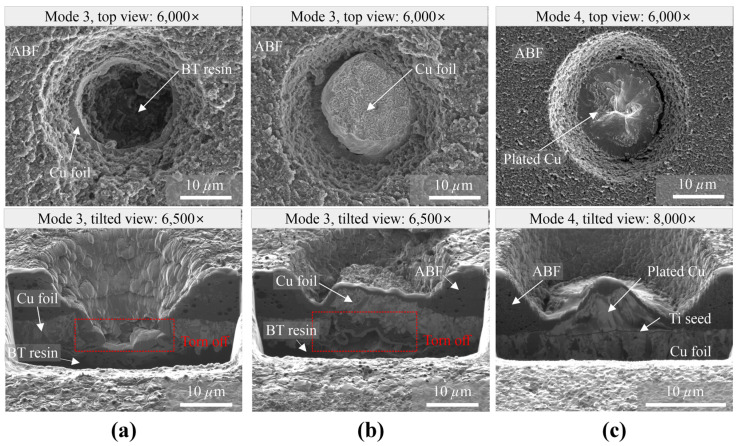
SEM micrographs of (**a**,**b**) Mode 3 and (**c**) Mode 4 after QVP testing, showing stable interfacial bonding.

Further evaluation using SEM indicates that most fracture events occurred either at the via bottom (Mode 3), as shown in Figure 13a,b, or within the dielectric layer (Mode 4), as shown in Figure 13c, suggesting that interfacial failure was effectively suppressed. Tilted SEM micrographs further revealed that the fracture surface was primarily located at the via bottom without any observable delamination at the interface between the bottom Cu foil and the electroplated Cu, confirming the formation of a robust metallurgical bond. This behavior can be attributed to the presence of the Cu SMBL, which effectively prevented debris redeposition during laser drilling and maintained a clean via-bottom surface. In addition, the SMBL facilitated efficient transport of reactive plasma species during the desmearing process, enabling thorough removal of residual debris and ensuring proper surface activation at the via bottom. As a result, a defect-free and well-bonded interface was achieved, leading to improved interfacial reliability and stable vertical interconnect performance. The absence of interfacial delamination and the predominance of cohesive failure modes demonstrated that the SMBL-assisted process fundamentally enhanced interfacial integrity by ensuring a clean and well-activated via bottom during both laser drilling and plasma desmear.

The QVP results were compared with those of the conventional process, as shown in Table 1. The control (without SMBL) exhibited a higher peel strength of 0.62 kgf/cm, which is attributed to mechanical interlocking caused by the rough dielectric surface (*R*_a_ ≈ 0.12 μm). However, such surface roughness introduces significant process limitations. During plasma desmear, the via opening tends to widen uncontrollably, making it difficult to form vias smaller than 10 μm. In addition, the rough surface disrupts fine circuit patterning by reducing dimensional accuracy and causing nonuniform interfaces. By contrast, the SMBL-assisted process with a 100 nm Cu layer achieved a stable peel strength of 0.46 kgf/cm even after the QVP test, despite the much smoother surface (*R*_a_ ≈ 0.04 μm). The adhesion decreased by only approximately 7% from the initial value, indicating that the bonding remained stable under mechanical stress. These results indicate that strong adhesion does not require a rough surface and suggest that robust interfacial bonding can be achieved even at low surface roughness levels. Instead, the SMBL preserved the dielectric surface in a smooth state and facilitated the formation of a clean and uniform bonding interface. The Ti/Cu seed layer promoted chemical bonding at the interface, whereas the smooth surface ensured intimate and continuous contact between the metal and dielectric. At the same time, the via shape was well maintained, and process control was improved. This approach reduced defects caused by roughness and enabled precise via formation, providing a practical solution for fine-pitch microvias and low-loss interconnects in next-generation high-frequency packaging.

## 4. Conclusions

This study developed a manufacturing-compatible ns-UV laser microvia process using a Cu SMBL to enable fine via formation while preserving the dielectric surface, addressing the critical challenge of simultaneously achieving sub-10 µm scaling and nanoscale surface smoothness in advanced packaging substrates. The Cu SMBL blocked debris redeposition during laser drilling and shielded the dielectric from ion bombardment during plasma desmear, preventing surface roughening. By controlling laser power, shot count, and SMBL thickness, sub-10 μm microvias (~8 μm) were formed in 10 μm thick ABF. The Cu SMBL distributed heat away from the irradiation zone, reduced the heat-affected zone, and limited lateral widening at the via entrance, resulting in improved via shape and a higher taper.

The dielectric surface remained nearly unchanged after processing (*R*_a_: 6.36 → 6.43 nm, Δ*R*_a_ = 0.07 nm), whereas the absence of an SMBL resulted in increased roughness to >80 nm. The smooth interface provided by the SMBL mitigates current scattering at high frequency, where the skin depth is on the order of hundreds of nanometers. Reliable interconnects were achieved on the smooth surface, with adhesion of 0.46 kgf/cm after QVP, remaining within the acceptable range without mechanical interlocking. Most vias failed through Mode 3 or 4, and SEM micrographs confirmed a debris-free, delamination-free interface at the via bottom.

The SMBL maintained surface integrity while controlling lateral via expansion, enabling fine-pitch RDL and low-loss, high-frequency packaging. Unlike conventional CO_2_ or wet desmearing processes that degrade surface morphology, this approach preserved nanoscale smoothness while achieving aggressive via scaling using industry-relevant ns-UV laser systems. This process provides a practical pathway for implementing sub-10 μm interconnects required for next-generation heterogeneous integration and high-I/O-density substrates operating at tens of GHz while preserving dielectric surface roughness at approximately 6 nm.

A systematic investigation of the critical thickness of the SMBL, particularly its quantitative effects on via dimensional accuracy and laser energy efficiency, is an important direction for future research.

## Figures and Tables

**Table 1:** Comparison of copper layer properties and reliability after QVP.

Parameters	w/ SMBL	w/o SMBL
Cu SMBL thickness (nm)	100	0
Cure condition (Step 1)	100~120 °C/30 min	
Cure condition (Step 2)	180~185 °C/30 min	
Ti/Cu Sputter	Ti 100 nm/Cu 300 nm	
Cu electroplating	Thickness of 20 μm and annealing @ 190 °C for 60 min	
90° peel strength (kgf/cm)	0.46	0.62
Surface roughness, *R*_a_ (μm)	0.04	0.12
Surface roughness, *R*_z_ (μm)	0.20	1.03

## Data Availability

The original contributions presented in this study are included in the article. Further inquiries can be directed to the corresponding author.

## Abbreviations

The following abbreviations are used in this manuscript:

ABF	Ajinomoto build-up film
AFM	Atomic force microscopy
BT	Bismaleimide triazine
DC	Direct current
DI	Deionized water
FIB	Focused ion beam
L/S	Line/space
HAZ	Heat-affected zone
I/O	Input/output
Ns-UV	Nanosecond ultraviolet
OM	Optical microscopy
QVP	Quick via pull
SEM	Scanning electron microscopy
SI	Signal integrity
SMBL	Sacrificial metal barrier layer
UV	Ultraviolet

## References

[1] Waldrop M.M. (2016). The chips are down for Moore’s law. Nature.

[2] Thompson S.E., Parthasarathy S. (2006). Moore’s law: The future of Si microelectronics. Mater. Today.

[3] Lau J.H. (2023). Recent advances and trends in multiple system and heterogeneous integration with TSV interposers. IEEE Trans. Compon. Packag. Manuf. Technol..

[4] Okamoto D., Shibasaki Y., Shibata D., Hanada T. (2018). New photosensitive dielectric material for high-density RDL with ultra-small photo-vias and high reliability. Int. Symp. Microelectron..

[5] Liu F., Zhang R., DeProspo B.H., Dwarakanath S., Nimbalkar P., Ravichandran S., Weyers D., Kathaperumal M., Tummala R.R., Swaminathan M. (2020). Advances in high performance RDL technologies for enabling IO density of 500 IOs/mm/layer and 8-μm IO pitch using low-k dielectrics. Proceedings of the 70th IEEE Electronic Components and Technology Conference (ECTC), Orlando, FL, USA.

[6] Liang C.L., Lin Y.S., Kao C.L., Tarng D., Wang S.B., Hung Y.C., Lin G.T., Lin K.L. (2020). Electromigration reliability of advanced high-density fan-out packaging with fine-pitch 2-/2-μm L/S Cu redistribution lines. IEEE Trans. Compon. Packag. Manuf. Technol..

[7] Kim J., Jaehoon C., Sanguk K., Jooyoung C., Yongjin P., Gyoungbum K., Sangyu K., Sangwook P., Hwasub O., Won L.S. (2021). Cost effective 2.3 D packaging solution by using fanout panel level RDL. 71st Electronic Components and Technology Conference (ECTC).

[8] Zhang Y., Tian W., Wang H., Wang L., Yang Z., Shao W., Chen Z., Zhou B. (2024). High-frequency transmission characteristic analysis of TSV-RDL interconnects. IEEE Trans. Compon. Packag. Manuf. Technol..

[9] Dwarakanath S., Raj P.M., Agarwal A., Okamoto D., Kubo A., Liu F., Kathaperumal M., Tummala R.R. Evaluation of fine-pitch routing capabilities of advanced dielectric materials for high speed panel-RDL in 2.5 D interposer and fan-out packages. Proceedings of the 69th IEEE Electronic Components and Technology Conference (ECTC).

[10] Shao Y., Peng Z., Lee J.F. (2012). Signal integrity analysis of high-speed interconnects by using nonconformal domain decomposition method. IEEE Trans. Compon. Packag. Manuf. Technol..

[11] Shimizu A., Fukada K., Abe M., Matsui A., Endo S. (2025). Evaluation of the high-frequency transmission characteristics of coplanar transmission lines fabricated on a copper-plated cycloolefin polymer using a direct sputtering copper seed layer. IEEE Access.

[12] Hinaga S., Koledintseva M.Y., Anmula P.K.R., Drewniak J.L. Effect of conductor surface roughness upon measured loss and extracted values of PCB laminate material dissipation factor. Proceedings of the IPC.

[13] He X., Huang L., Xiao M., Yu C., Li E., Shao W. (2024). Investigation on the new reliability issues of PCB in 5G millimeter wave application. Microelectron. Int..

[14] Chiang Y.C., Chang Y.H., Yang Z.Y., Yu C.J., Chou W.L., Ho C.E. (2025). Enhancing the high-frequency signal performance through surface morphological modification of Cu interconnects. Measurement.

[15] Curran B., Ndip I., Guttowski S., Reichl H. (2010). A methodology for combined modeling of skin, proximity, edge, and surface roughness effects. IEEE Trans. Microw. Theory Tech..

[16] Devahif T. Ultra low profile copper foil for very low loss material. Proceedings of the SMTA International.

[17] Watanabe A.O., Kanno K., Ito H., Tummala R.R., Swaminathan M. (2021). High-density low-loss millimeter-wave package interconnects with the impact of dielectric-material surface roughness. Appl. Phys. Lett..

[18] Imani R., Chouhan S., Putaala J., Nousiainen O., Hagberg J., Myllymäki S., Acharya S., Jantunen H., Delsing J. A fully additive fabrication approach for sub-10-Micrometer microvia suitable for 3-D system-in-package integration. Proceedings of the IEEE 73rd Electronic Components and Technology Conference (ECTC).

[19] Endo S., Habu T., Kikuiri H., Aiba A., Miura M., Horibe H., Suzuki H., Yabu S. New desmear process of organic substrate applications by Photodesmear technology. Proceedings of the 2017 12th International Microsystems, Packaging, Assembly and Circuits Technology Conference (IMPACT).

[20] Liu F., Zhang R., Khurana G., Deprospo B.H., Tummala R.R., Swaminathan M. (2020). Smaller microvias for packaging interconnects by picosecond UV laser with a nanometer metal barrier layer: A feasibility study. IEEE Trans. Compon. Packag. Manuf. Technol..

[21] Liu F., Khurana G., Zhang R., Watanabe A., DeProspo B.H., Nair C., Swaminathan M. (2019). Innovative sub-5um microvias by picosecond UV laser for post-Moore packaging interconnects. IEEE Trans. Compon. Packag. Manuf. Technol..

[22] Franz D., Häfner T., Kunz T., Roth G.-L., Rung S., Esen C., Hellmann R. (2022). Ultrashort pulsed laser drilling of printed circuit board materials. Materials.

[23] Franz D., Häfner T., Bischoff K., Helm J., Kunz T., Rung S., Hellmann R. (2023). Picosecond laser microvia drilling of ABF material using MHz burst mode. Mater. Res. Express.

[24] Park N.S., Lee T.Y., Kim K.M., Lee S.Y., Yoon S., You M.S., Yoo G., Yoo S. (2025). Technology for Forming Micro Vias Smaller Than 20 μm with Low Surface Roughness on Build-Up Films Using UV Nanosecond Laser Drilling and Plasma Desmearing. IEEE 75th Electronic Components and Technology Conference (ECTC).

[25] (1997). Geometrical Product Specifications (GPS)—Surface Texture: Profile Method—Terms, Definitions and Surface Texture Parameters.

[26] (2021). Geometrical Product Specifications (GPS)—Surface Texture: Areal—Part 2: Terms, Definitions and Surface Texture Parameters.

[27] Park N.S., Lee T.Y., Park D., Jo M., Kim S., Yoo S., You M.S., Lee G., Kim K.M. (2025). Low-roughness UV laser drilling for Sub-10 μm Vias in advanced semiconductor package substrates. 20th International Microsystems, Packaging, Assembly and Circuits Technology Conference (Impact).

[28] Morikawa Y., Sato M., Murayama T., Fujinaga T. (2018). Fabrication of fine via and line/space in low CTE film for panel fan-out using a dry etching technology. 68th Electronic Components and Technology Conference (ECTC).

